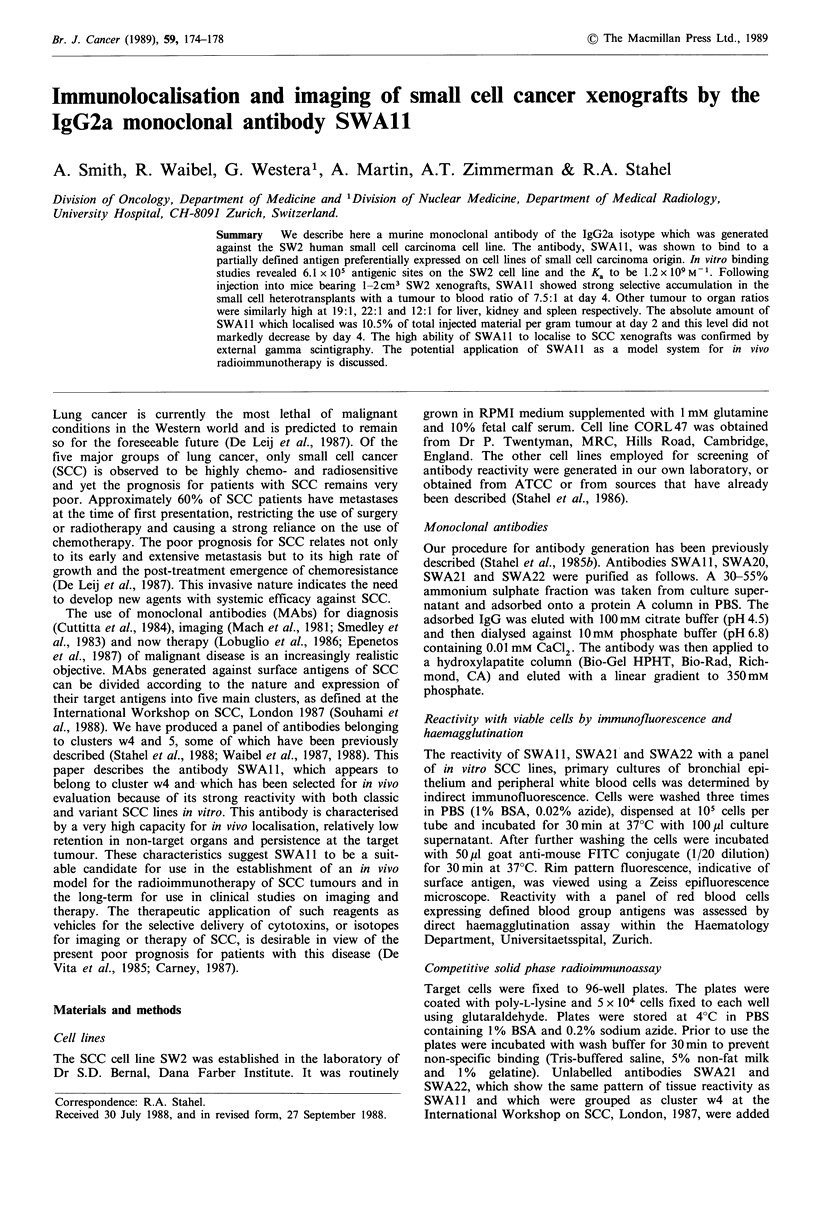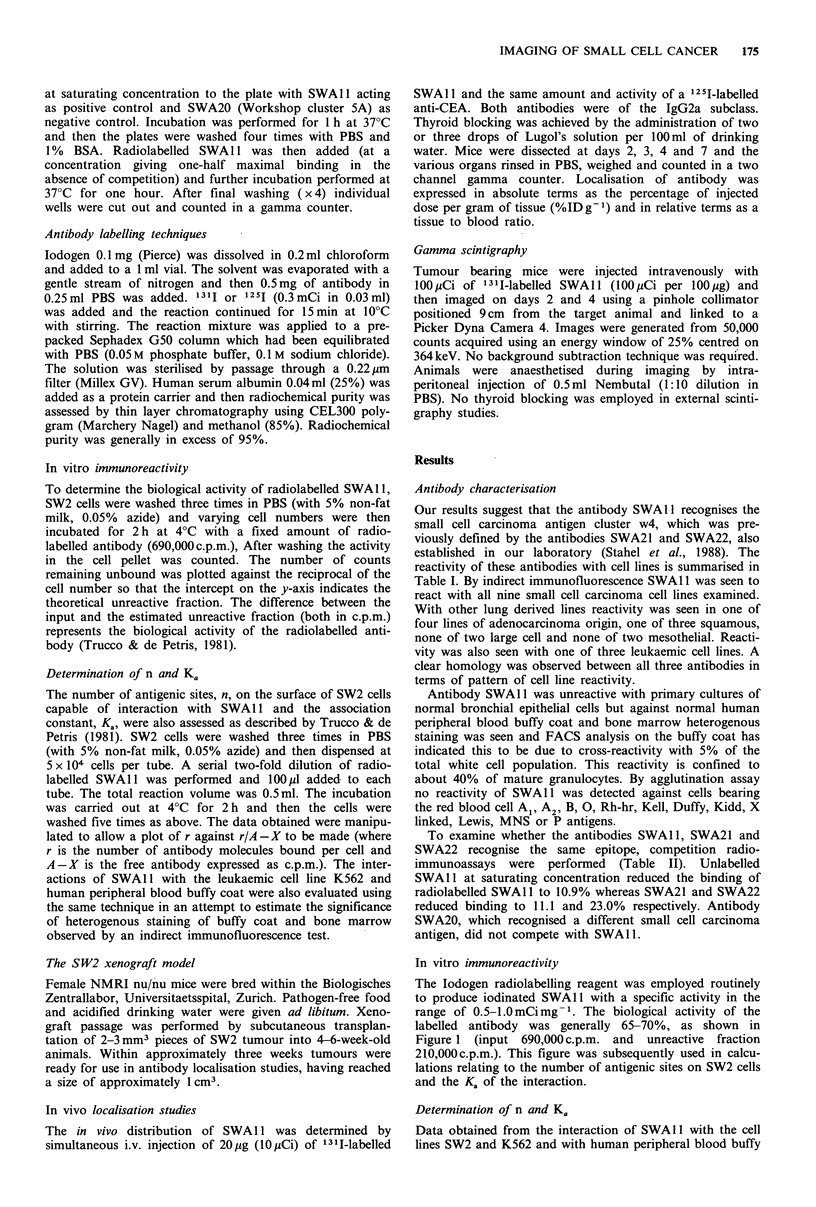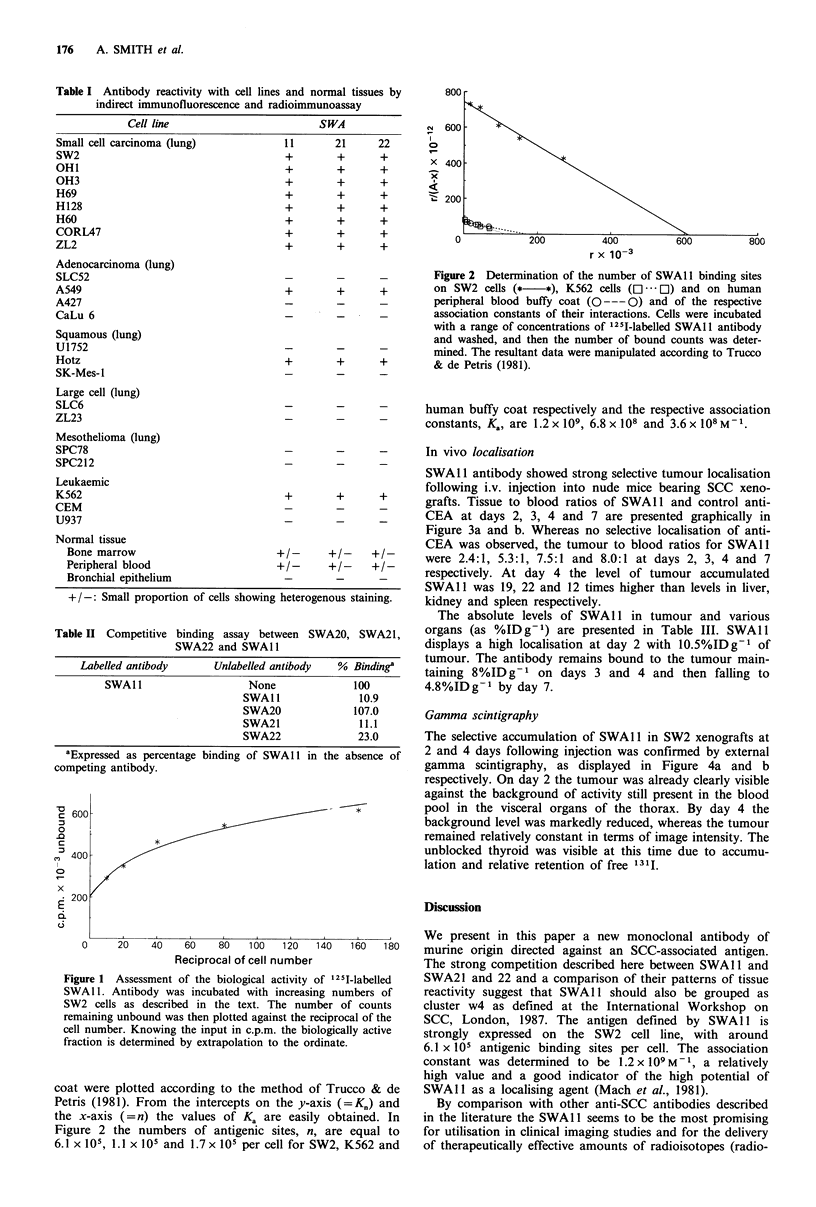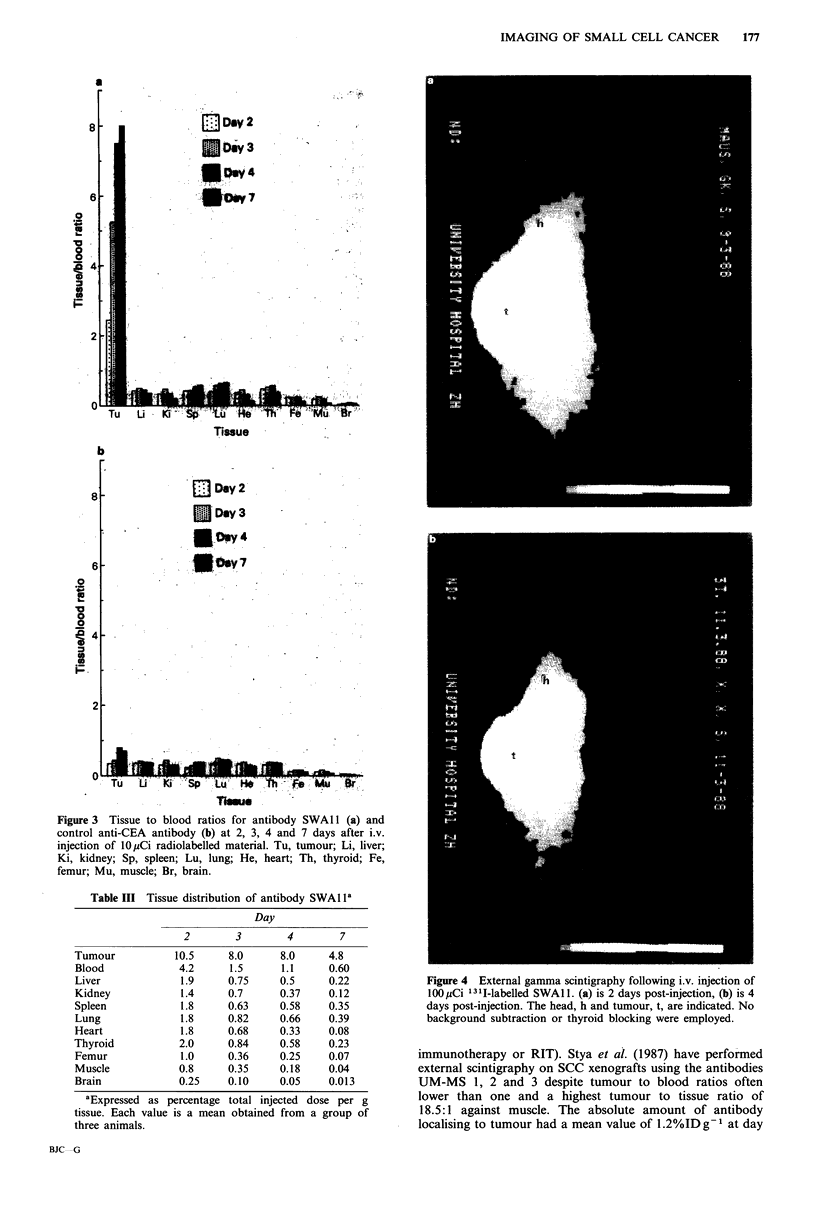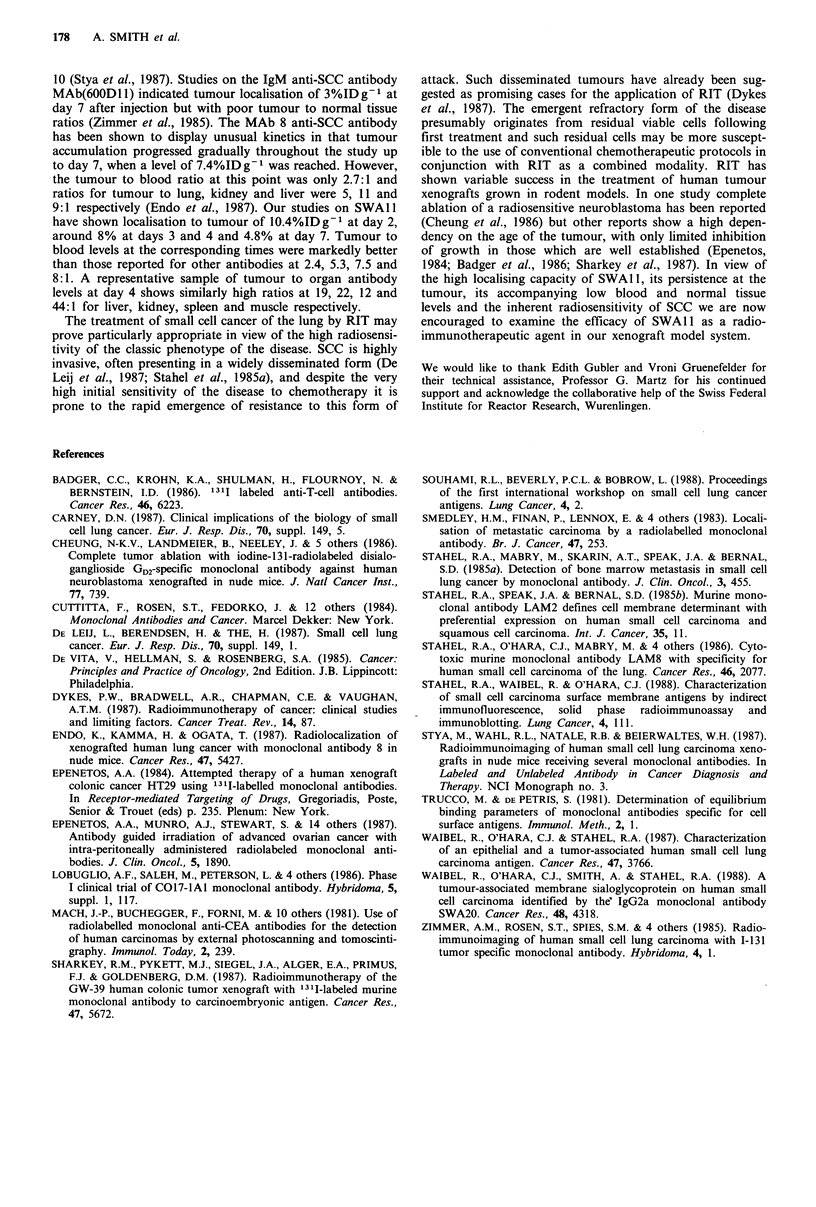# Immunolocalisation and imaging of small cell cancer xenografts by the IgG2a monoclonal antibody SWA11.

**DOI:** 10.1038/bjc.1989.36

**Published:** 1989-02

**Authors:** A. Smith, R. Waibel, G. Westera, A. Martin, A. T. Zimmerman, R. A. Stahel

**Affiliations:** Department of Medicine, University Hospital, Zurich, Switzerland.

## Abstract

**Images:**


Br. J. Cancer (1989), 59, 174–178 © The Macmillan Press Ltd., 1989

### Immunolocalisation and imaging of small cell cancer xenografts by the IgG2a monoclonal antibody SWA11

A. Smith, R. Waibel, G. Westera[1], A. Martin, A.T. Zimmerman & R.A. Stahel

*Division of Oncology, Department of Medicine and [1]Division of Nuclear Medicine, Department of Medical Radiology, University Hospital, CH-8091 Zurich, Switzerland.*

**Summary** We describe here a murine monoclonal antibody of the IgG2a isotype which was generated against the SW2 human small cell carcinoma cell line. The antibody, SWA11, was shown to bind to a partially defined antigen preferentially expressed on cell lines of small cell carcinoma origin. *In vitro* binding studies revealed $6.1 \times 10^5$ antigenic sites on the SW2 cell line and the $K_a$ to be $1.2 \times 10^9 \text{M}^{-1}$. Following injection into mice bearing 1–2 cm$^3$ SW2 xenografts, SWA11 showed strong selective accumulation in the small cell heterotransplants with a tumour to blood ratio of 7.5:1 at day 4. Other tumour to organ ratios were similarly high at 19:1, 22:1 and 12:1 for liver, kidney and spleen respectively. The absolute amount of SWA11 which localised was 10.5% of total injected material per gram tumour at day 2 and this level did not markedly decrease by day 4. The high ability of SWA11 to localise to SCC xenografts was confirmed by external gamma scintigraphy. The potential application of SWA11 as a model system for *in vivo* radioimmunotherapy is discussed.

Lung cancer is currently the most lethal of malignant conditions in the Western world and is predicted to remain so for the foreseeable future (De Leij *et al.*, 1987). Of the five major groups of lung cancer, only small cell cancer (SCC) is observed to be highly chemo- and radiosensitive and yet the prognosis for patients with SCC remains very poor. Approximately 60% of SCC patients have metastases at the time of first presentation, restricting the use of surgery or radiotherapy and causing a strong reliance on the use of chemotherapy. The poor prognosis for SCC relates not only to its early and extensive metastasis but to its high rate of growth and the post-treatment emergence of chemoresistance (De Leij *et al.*, 1987). This invasive nature indicates the need to develop new agents with systemic efficacy against SCC.

The use of monoclonal antibodies (MAbs) for diagnosis (Cuttitta *et al.*, 1984), imaging (Mach *et al.*, 1981; Smedley *et al.*, 1983) and now therapy (Lobuglio *et al.*, 1986; Epenetos *et al.*, 1987) of malignant disease is an increasingly realistic objective. MAbs generated against surface antigens of SCC can be divided according to the nature and expression of their target antigens into five main clusters, as defined at the International Workshop on SCC, London 1987 (Souhami *et al.*, 1988). We have produced a panel of antibodies belonging to clusters w4 and 5, some of which have been previously described (Stahel *et al.*, 1988; Waibel *et al.*, 1987, 1988). This paper describes the antibody SWA11, which appears to belong to cluster w4 and which has been selected for *in vivo* evaluation because of its strong reactivity with both classic and variant SCC lines *in vitro*. This antibody is characterised by a very high capacity for *in vivo* localisation, relatively low retention in non-target organs and persistence at the target tumour. These characteristics suggest SWA11 to be a suitable candidate for use in the establishment of an *in vivo* model for the radioimmunotherapy of SCC tumours and in the long-term for use in clinical studies on imaging and therapy. The therapeutic application of such reagents as vehicles for the selective delivery of cytotoxins, or isotopes for imaging or therapy of SCC, is desirable in view of the present poor prognosis for patients with this disease (De Vita *et al.*, 1985; Carney, 1987).

### Materials and methods

### *Cell lines*

The SCC cell line SW2 was established in the laboratory of Dr S.D. Bernal, Dana Farber Institute. It was routinely grown in RPMI medium supplemented with 1 mM glutamine and 10% fetal calf serum. Cell line CORL 47 was obtained from Dr P. Twentyman, MRC, Hills Road, Cambridge, England. The other cell lines employed for screening of antibody reactivity were generated in our own laboratory, or obtained from ATCC or from sources that have already been described (Stahel *et al.*, 1986).

### *Monoclonal antibodies*

Our procedure for antibody generation has been previously described (Stahel *et al.*, 1985*b*). Antibodies SWA11, SWA20, SWA21 and SWA22 were purified as follows. A 30–55% ammonium sulphate fraction was taken from culture supernatant and adsorbed onto a protein A column in PBS. The adsorbed IgG was eluted with 100 mM citrate buffer (pH 4.5) and then dialysed against 10 mM phosphate buffer (pH 6.8) containing 0.01 mM $CaCl_2$. The antibody was then applied to a hydroxylapatite column (Bio-Gel HPHT, Bio-Rad, Richmond, CA) and eluted with a linear gradient to 350 mM phosphate.

### *Reactivity with viable cells by immunofluorescence and haemagglutination*

The reactivity of SWA11, SWA21 and SWA22 with a panel of *in vitro* SCC lines, primary cultures of bronchial epithelium and peripheral white blood cells was determined by indirect immunofluorescence. Cells were washed three times in PBS (1% BSA, 0.02% azide), dispensed at $10^5$ cells per tube and incubated for 30 min at 37°C with 100 $\mu$l culture supernatant. After further washing the cells were incubated with 50 $\mu$l goat anti-mouse FITC conjugate (1/20 dilution) for 30 min at 37°C. Rim pattern fluorescence, indicative of surface antigen, was viewed using a Zeiss epifluorescence microscope. Reactivity with a panel of red blood cells expressing defined blood group antigens was assessed by direct haemagglutination assay within the Haematology Department, Universitaetsspital, Zurich.

### *Competitive solid phase radioimmunoassay*

Target cells were fixed to 96-well plates. The plates were coated with poly-L-lysine and $5 \times 10^4$ cells fixed to each well using glutaraldehyde. Plates were stored at 4°C in PBS containing 1% BSA and 0.2% sodium azide. Prior to use the plates were incubated with wash buffer for 30 min to prevent non-specific binding (Tris-buffered saline, 5% non-fat milk and 1% gelatine). Unlabelled antibodies SWA21 and SWA22, which show the same pattern of tissue reactivity as SWA11 and which were grouped as cluster w4 at the International Workshop on SCC, London, 1987, were added

Correspondence: R.A. Stahel.
Received 30 July 1988, and in revised form, 27 September 1988.

at saturating concentration to the plate with SWA11 acting as positive control and SWA20 (Workshop cluster 5A) as negative control. Incubation was performed for 1 h at 37°C and then the plates were washed four times with PBS and 1% BSA. Radiolabelled SWA11 was then added (at a concentration giving one-half maximal binding in the absence of competition) and further incubation performed at 37°C for one hour. After final washing (×4) individual wells were cut out and counted in a gamma counter.

*Antibody labelling techniques*

Iodogen 0.1 mg (Pierce) was dissolved in 0.2 ml chloroform and added to a 1 ml vial. The solvent was evaporated with a gentle stream of nitrogen and then 0.5 mg of antibody in 0.25 ml PBS was added. $^{131}I$ or $^{125}I$ (0.3 mCi in 0.03 ml) was added and the reaction continued for 15 min at 10°C with stirring. The reaction mixture was applied to a pre-packed Sephadex G50 column which had been equilibrated with PBS (0.05 M phosphate buffer, 0.1 M sodium chloride). The solution was sterilised by passage through a 0.22 $\mu$m filter (Millex GV). Human serum albumin 0.04 ml (25%) was added as a protein carrier and then radiochemical purity was assessed by thin layer chromatography using CEL300 polygram (Marchery Nagel) and methanol (85%). Radiochemical purity was generally in excess of 95%.

In vitro *immunoreactivity*

To determine the biological activity of radiolabelled SWA11, SW2 cells were washed three times in PBS (with 5% non-fat milk, 0.05% azide) and varying cell numbers were then incubated for 2 h at 4°C with a fixed amount of radiolabelled antibody (690,000 c.p.m.), After washing the activity in the cell pellet was counted. The number of counts remaining unbound was plotted against the reciprocal of the cell number so that the intercept on the $y$-axis indicates the theoretical unreactive fraction. The difference between the input and the estimated unreactive fraction (both in c.p.m.) represents the biological activity of the radiolabelled antibody (Trucco & de Petris, 1981).

*Determination of* n *and* $K_a$

The number of antigenic sites, $n$, on the surface of SW2 cells capable of interaction with SWA11 and the association constant, $K_a$, were also assessed as described by Trucco & de Petris (1981). SW2 cells were washed three times in PBS (with 5% non-fat milk, 0.05% azide) and then dispensed at $5 \times 10^4$ cells per tube. A serial two-fold dilution of radiolabelled SWA11 was performed and 100 $\mu$l added to each tube. The total reaction volume was 0.5 ml. The incubation was carried out at 4°C for 2 h and then the cells were washed five times as above. The data obtained were manipulated to allow a plot of $r$ against $r/A-X$ to be made (where $r$ is the number of antibody molecules bound per cell and $A-X$ is the free antibody expressed as c.p.m.). The interactions of SWA11 with the leukaemic cell line K562 and human peripheral blood buffy coat were also evaluated using the same technique in an attempt to estimate the significance of heterogenous staining of buffy coat and bone marrow observed by an indirect immunofluorescence test.

*The SW2 xenograft model*

Female NMRI nu/nu mice were bred within the Biologisches Zentrallabor, Universitaetsspital, Zurich. Pathogen-free food and acidified drinking water were given *ad libitum*. Xenograft passage was performed by subcutaneous transplantation of 2–3 mm³ pieces of SW2 tumour into 4–6-week-old animals. Within approximately three weeks tumours were ready for use in antibody localisation studies, having reached a size of approximately 1 cm³.

In vivo *localisation studies*

The *in vivo* distribution of SWA11 was determined by simultaneous i.v. injection of 20 $\mu$g (10 $\mu$Ci) of $^{131}I$-labelled SWA11 and the same amount and activity of a $^{125}I$-labelled anti-CEA. Both antibodies were of the IgG2a subclass. Thyroid blocking was achieved by the administration of two or three drops of Lugol's solution per 100 ml of drinking water. Mice were dissected at days 2, 3, 4 and 7 and the various organs rinsed in PBS, weighed and counted in a two channel gamma counter. Localisation of antibody was expressed in absolute terms as the percentage of injected dose per gram of tissue (%ID $g^{-1}$) and in relative terms as a tissue to blood ratio.

*Gamma scintigraphy*

Tumour bearing mice were injected intravenously with 100 $\mu$Ci of $^{131}I$-labelled SWA11 (100 $\mu$Ci per 100 $\mu$g) and then imaged on days 2 and 4 using a pinhole collimator positioned 9 cm from the target animal and linked to a Picker Dyna Camera 4. Images were generated from 50,000 counts acquired using an energy window of 25% centred on 364 keV. No background subtraction technique was required. Animals were anaesthetised during imaging by intraperitoneal injection of 0.5 ml Nembutal (1:10 dilution in PBS). No thyroid blocking was employed in external scintigraphy studies.

### Results

*Antibody characterisation*

Our results suggest that the antibody SWA11 recognises the small cell carcinoma antigen cluster w4, which was previously defined by the antibodies SWA21 and SWA22, also established in our laboratory (Stahel *et al.*, 1988). The reactivity of these antibodies with cell lines is summarised in Table I. By indirect immunofluorescence SWA11 was seen to react with all nine small cell carcinoma cell lines examined. With other lung derived lines reactivity was seen in one of four lines of adenocarcinoma origin, one of three squamous, none of two large cell and none of two mesothelial. Reactivity was also seen with one of three leukaemic cell lines. A clear homology was observed between all three antibodies in terms of pattern of cell line reactivity.

Antibody SWA11 was unreactive with primary cultures of normal bronchial epithelial cells but against normal human peripheral blood buffy coat and bone marrow heterogenous staining was seen and FACS analysis on the buffy coat has indicated this to be due to cross-reactivity with 5% of the total white cell population. This reactivity is confined to about 40% of mature granulocytes. By agglutination assay no reactivity of SWA11 was detected against cells bearing the red blood cell $A_1$, $A_2$, B, O, Rh-hr, Kell, Duffy, Kidd, X linked, Lewis, MNS or P antigens.

To examine whether the antibodies SWA11, SWA21 and SWA22 recognise the same epitope, competition radioimmunoassays were performed (Table II). Unlabelled SWA11 at saturating concentration reduced the binding of radiolabelled SWA11 to 10.9% whereas SWA21 and SWA22 reduced binding to 11.1 and 23.0% respectively. Antibody SWA20, which recognised a different small cell carcinoma antigen, did not compete with SWA11.

In vitro *immunoreactivity*

The Iodogen radiolabelling reagent was employed routinely to produce iodinated SWA11 with a specific activity in the range of 0.5–1.0 mCi $mg^{-1}$. The biological activity of the labelled antibody was generally 65–70%, as shown in Figure 1 (input 690,000 c.p.m. and unreactive fraction 210,000 c.p.m.). This figure was subsequently used in calculations relating to the number of antigenic sites on SW2 cells and the $K_a$ of the interaction.

*Determination of* n *and* $K_a$

Data obtained from the interaction of SWA11 with the cell lines SW2 and K562 and with human peripheral blood buffy

**Table I** Antibody reactivity with cell lines and normal tissues by indirect immunofluorescence and radioimmunoassay

| *Cell line* | *SWA* | | |
|---|---|---|---|
| Small cell carcinoma (lung) | 11 | 21 | 22 |
| SW2 | + | + | + |
| OH1 | + | + | + |
| OH3 | + | + | + |
| H69 | + | + | + |
| H128 | + | + | + |
| H60 | + | + | + |
| CORL47 | + | + | + |
| ZL2 | + | + | + |
| Adenocarcinoma (lung) | | | |
| SLC52 | − | − | − |
| A549 | + | + | + |
| A427 | − | − | − |
| CaLu 6 | − | − | − |
| Squamous (lung) | | | |
| U1752 | − | − | − |
| Hotz | + | + | + |
| SK-Mes-1 | − | − | − |
| Large cell (lung) | | | |
| SLC6 | − | − | − |
| ZL23 | − | − | − |
| Mesothelioma (lung) | | | |
| SPC78 | − | − | − |
| SPC212 | − | − | − |
| Leukaemic | | | |
| K562 | + | + | + |
| CEM | − | − | − |
| U937 | − | − | − |
| Normal tissue | | | |
| Bone marrow | +/− | +/− | +/− |
| Peripheral blood | +/− | +/− | +/− |
| Bronchial epithelium | − | − | − |

+/−: Small proportion of cells showing heterogenous staining.

**Table II** Competitive binding assay between SWA20, SWA21, SWA22 and SWA11

| *Labelled antibody* | *Unlabelled antibody* | *% Binding*[a] |
|---|---|---|
| SWA11 | None | 100 |
| | SWA11 | 10.9 |
| | SWA20 | 107.0 |
| | SWA21 | 11.1 |
| | SWA22 | 23.0 |

[a]Expressed as percentage binding of SWA11 in the absence of competing antibody.

**Figure 1** Assessment of the biological activity of $^{125}$I-labelled SWA11. Antibody was incubated with increasing numbers of SW2 cells as described in the text. The number of counts remaining unbound was then plotted against the reciprocal of the cell number. Knowing the input in c.p.m. the biologically active fraction is determined by extrapolation to the ordinate.

coat were plotted according to the method of Trucco & de Petris (1981). From the intercepts on the *y*-axis ($=K_n$) and the *x*-axis ($=n$) the values of $K_a$ are easily obtained. In Figure 2 the numbers of antigenic sites, *n*, are equal to $6.1\times10^5$, $1.1\times10^5$ and $1.7\times10^5$ per cell for SW2, K562 and

**Figure 2** Determination of the number of SWA11 binding sites on SW2 cells (*——*), K562 cells (□···□) and on human peripheral blood buffy coat (○---○) and of the respective association constants of their interactions. Cells were incubated with a range of concentrations of $^{125}$I-labelled SWA11 antibody and washed, and then the number of bound counts was determined. The resultant data were manipulated according to Trucco & de Petris (1981).

human buffy coat respectively and the respective association constants, $K_a$, are $1.2\times10^9$, $6.8\times10^8$ and $3.6\times10^8\,\mathrm{M}^{-1}$.

### In vivo *localisation*

SWA11 antibody showed strong selective tumour localisation following i.v. injection into nude mice bearing SCC xenografts. Tissue to blood ratios of SWA11 and control anti-CEA at days 2, 3, 4 and 7 are presented graphically in Figure 3a and b. Whereas no selective localisation of anti-CEA was observed, the tumour to blood ratios for SWA11 were 2.4:1, 5.3:1, 7.5:1 and 8.0:1 at days 2, 3, 4 and 7 respectively. At day 4 the level of tumour accumulated SWA11 was 19, 22 and 12 times higher than levels in liver, kidney and spleen respectively.

The absolute levels of SWA11 in tumour and various organs (as $\%\mathrm{ID\,g}^{-1}$) are presented in Table III. SWA11 displays a high localisation at day 2 with $10.5\%\mathrm{ID\,g}^{-1}$ of tumour. The antibody remains bound to the tumour maintaining $8\%\mathrm{ID\,g}^{-1}$ on days 3 and 4 and then falling to $4.8\%\mathrm{ID\,g}^{-1}$ by day 7.

### *Gamma scintigraphy*

The selective accumulation of SWA11 in SW2 xenografts at 2 and 4 days following injection was confirmed by external gamma scintigraphy, as displayed in Figure 4a and b respectively. On day 2 the tumour was already clearly visible against the background of activity still present in the blood pool in the visceral organs of the thorax. By day 4 the background level was markedly reduced, whereas the tumour remained relatively constant in terms of image intensity. The unblocked thyroid was visible at this time due to accumulation and relative retention of free $^{131}$I.

### Discussion

We present in this paper a new monoclonal antibody of murine origin directed against an SCC-associated antigen. The strong competition described here between SWA11 and SWA21 and 22 and a comparison of their patterns of tissue reactivity suggest that SWA11 should also be grouped as cluster w4 as defined at the International Workshop on SCC, London, 1987. The antigen defined by SWA11 is strongly expressed on the SW2 cell line, with around $6.1\times10^5$ antigenic binding sites per cell. The association constant was determined to be $1.2\times10^9\,\mathrm{M}^{-1}$, a relatively high value and a good indicator of the high potential of SWA11 as a localising agent (Mach *et al.*, 1981).

By comparison with other anti-SCC antibodies described in the literature the SWA11 seems to be the most promising for utilisation in clinical imaging studies and for the delivery of therapeutically effective amounts of radioisotopes (radio-

**Figure 3** Tissue to blood ratios for antibody SWA11 (**a**) and control anti-CEA antibody (**b**) at 2, 3, 4 and 7 days after i.v. injection of 10 $\mu$Ci radiolabelled material. Tu, tumour; Li, liver; Ki, kidney; Sp, spleen; Lu, lung; He, heart; Th, thyroid; Fe, femur; Mu, muscle; Br, brain.

**Table III** Tissue distribution of antibody SWA11[a]

| | *Day* | | | |
|---|---|---|---|---|
| | *2* | *3* | *4* | *7* |
| Tumour | 10.5 | 8.0 | 8.0 | 4.8 |
| Blood | 4.2 | 1.5 | 1.1 | 0.60 |
| Liver | 1.9 | 0.75 | 0.5 | 0.22 |
| Kidney | 1.4 | 0.7 | 0.37 | 0.12 |
| Spleen | 1.8 | 0.63 | 0.58 | 0.35 |
| Lung | 1.8 | 0.82 | 0.66 | 0.39 |
| Heart | 1.8 | 0.68 | 0.33 | 0.08 |
| Thyroid | 2.0 | 0.84 | 0.58 | 0.23 |
| Femur | 1.0 | 0.36 | 0.25 | 0.07 |
| Muscle | 0.8 | 0.35 | 0.18 | 0.04 |
| Brain | 0.25 | 0.10 | 0.05 | 0.013 |

[a]Expressed as percentage total injected dose per g tissue. Each value is a mean obtained from a group of three animals.

**Figure 4** External gamma scintigraphy following i.v. injection of 100 $\mu$Ci $^{131}$I-labelled SWA11. (**a**) is 2 days post-injection, (**b**) is 4 days post-injection. The head, h and tumour, t, are indicated. No background subtraction or thyroid blocking were employed.

immunotherapy or RIT). Stya *et al.* (1987) have performed external scintigraphy on SCC xenografts using the antibodies UM-MS 1, 2 and 3 despite tumour to blood ratios often lower than one and a highest tumour to tissue ratio of 18.5:1 against muscle. The absolute amount of antibody localising to tumour had a mean value of 1.2%ID g$^{-1}$ at day

10 (Stya et al., 1987). Studies on the IgM anti-SCC antibody MAb(600D11) indicated tumour localisation of 3%ID $g^{-1}$ at day 7 after injection but with poor tumour to normal tissue ratios (Zimmer et al., 1985). The MAb 8 anti-SCC antibody has been shown to display unusual kinetics in that tumour accumulation progressed gradually throughout the study up to day 7, when a level of 7.4%ID $g^{-1}$ was reached. However, the tumour to blood ratio at this point was only 2.7:1 and ratios for tumour to lung, kidney and liver were 5, 11 and 9:1 respectively (Endo et al., 1987). Our studies on SWA11 have shown localisation to tumour of 10.4%ID $g^{-1}$ at day 2, around 8% at days 3 and 4 and 4.8% at day 7. Tumour to blood levels at the corresponding times were markedly better than those reported for other antibodies at 2.4, 5.3, 7.5 and 8:1. A representative sample of tumour to organ antibody levels at day 4 shows similarly high ratios at 19, 22, 12 and 44:1 for liver, kidney, spleen and muscle respectively.

The treatment of small cell cancer of the lung by RIT may prove particularly appropriate in view of the high radiosensitivity of the classic phenotype of the disease. SCC is highly invasive, often presenting in a widely disseminated form (De Leij et al., 1987; Stahel et al., 1985a), and despite the very high initial sensitivity of the disease to chemotherapy it is prone to the rapid emergence of resistance to this form of attack. Such disseminated tumours have already been suggested as promising cases for the application of RIT (Dykes et al., 1987). The emergent refractory form of the disease presumably originates from residual viable cells following first treatment and such residual cells may be more susceptible to the use of conventional chemotherapeutic protocols in conjunction with RIT as a combined modality. RIT has shown variable success in the treatment of human tumour xenografts grown in rodent models. In one study complete ablation of a radiosensitive neuroblastoma has been reported (Cheung et al., 1986) but other reports show a high dependency on the age of the tumour, with only limited inhibition of growth in those which are well established (Epenetos, 1984; Badger et al., 1986; Sharkey et al., 1987). In view of the high localising capacity of SWA11, its persistence at the tumour, its accompanying low blood and normal tissue levels and the inherent radiosensitivity of SCC we are now encouraged to examine the efficacy of SWA11 as a radioimmunotherapeutic agent in our xenograft model system.

We would like to thank Edith Gubler and Vroni Gruenefelder for their technical assistance, Professor G. Martz for his continued support and acknowledge the collaborative help of the Swiss Federal Institute for Reactor Research, Wurenlingen.

### References

BADGER, C.C., KROHN, K.A., SHULMAN, H., FLOURNOY, N. & BERNSTEIN, I.D. (1986). $^{131}$I labeled anti-T-cell antibodies. *Cancer Res.*, **46**, 6223.

CARNEY, D.N. (1987). Clinical implications of the biology of small cell lung cancer. *Eur. J. Resp. Dis.*, **70**, suppl. 149, 5.

CHEUNG, N-K.V., LANDMEIER, B., NEELEY, J. & 5 others (1986). Complete tumor ablation with iodine-131-radiolabeled disialoganglioside $G_{D2}$-specific monoclonal antibody against human neuroblastoma xenografted in nude mice. *J. Natl Cancer Inst.*, **77**, 739.

CUTTITTA, F., ROSEN, S.T., FEDORKO, J. & 12 others (1984). *Monoclonal Antibodies and Cancer*. Marcel Dekker: New York.

DE LEIJ, L., BERENDSEN, H. & THE, H. (1987). Small cell lung cancer. *Eur. J. Resp. Dis.*, **70**, suppl. 149, 1.

DE VITA, V., HELLMAN, S. & ROSENBERG, S.A. (1985). *Cancer: Principles and Practice of Oncology*, 2nd Edition. J.B. Lippincott: Philadelphia.

DYKES, P.W., BRADWELL, A.R., CHAPMAN, C.E. & VAUGHAN, A.T.M. (1987). Radioimmunotherapy of cancer: clinical studies and limiting factors. *Cancer Treat. Rev.*, **14**, 87.

ENDO, K., KAMMA, H. & OGATA, T. (1987). Radiolocalization of xenografted human lung cancer with monoclonal antibody 8 in nude mice. *Cancer Res.*, **47**, 5427.

EPENETOS, A.A. (1984). Attempted therapy of a human xenograft colonic cancer HT29 using $^{131}$I-labelled monoclonal antibodies. In *Receptor-mediated Targeting of Drugs*, Gregoriadis, Poste, Senior & Trouet (eds) p. 235. Plenum: New York.

EPENETOS, A.A., MUNRO, A.J., STEWART, S. & 14 others (1987). Antibody guided irradiation of advanced ovarian cancer with intra-peritoneally administered radiolabeled monoclonal antibodies. *J. Clin. Oncol.*, **5**, 1890.

LOBUGLIO, A.F., SALEH, M., PETERSON, L. & 4 others (1986). Phase I clinical trial of CO17-1A1 monoclonal antibody. *Hybridoma*, **5**, suppl. 1, 117.

MACH, J.-P., BUCHEGGER, F., FORNI, M. & 10 others (1981). Use of radiolabelled monoclonal anti-CEA antibodies for the detection of human carcinomas by external photoscanning and tomoscintigraphy. *Immunol. Today*, **2**, 239.

SHARKEY, R.M., PYKETT, M.J., SIEGEL, J.A., ALGER, E.A., PRIMUS, F.J. & GOLDENBERG, D.M. (1987). Radioimmunotherapy of the GW-39 human colonic tumor xenograft with $^{131}$I-labeled murine monoclonal antibody to carcinoembryonic antigen. *Cancer Res.*, **47**, 5672.

SOUHAMI, R.L., BEVERLY, P.C.L. & BOBROW, L. (1988). Proceedings of the first international workshop on small cell lung cancer antigens. *Lung Cancer*, **4**, 2.

SMEDLEY, H.M., FINAN, P., LENNOX, E. & 4 others (1983). Localisation of metastatic carcinoma by a radiolabelled monoclonal antibody. *Br. J. Cancer*, **47**, 253.

STAHEL, R.A., MABRY, M., SKARIN, A.T., SPEAK, J.A. & BERNAL, S.D. (1985a). Detection of bone marrow metastasis in small cell lung cancer by monoclonal antibody. *J. Clin. Oncol.*, **3**, 455.

STAHEL, R.A., SPEAK, J.A. & BERNAL, S.D. (1985b). Murine monoclonal antibody LAM2 defines cell membrane determinant with preferential expression on human small cell carcinoma and squamous cell carcinoma. *Int. J. Cancer*, **35**, 11.

STAHEL, R.A., O'HARA, C.J., MABRY, M. & 4 others (1986). Cytotoxic murine monoclonal antibody LAM8 with specificity for human small cell carcinoma of the lung. *Cancer Res.*, **46**, 2077.

STAHEL, R.A., WAIBEL, R. & O'HARA, C.J. (1988). Characterization of small cell carcinoma surface membrane antigens by indirect immunofluorescence, solid phase radioimmunoassay and immunoblotting. *Lung Cancer*, **4**, 111.

STYA, M., WAHL, R.L., NATALE, R.B. & BEIERWALTES, W.H. (1987). Radioimmunoimaging of human small cell lung carcinoma xenografts in nude mice receiving several monoclonal antibodies. In *Labeled and Unlabeled Antibody in Cancer Diagnosis and Therapy*. NCI Monograph no. 3.

TRUCCO, M. & DE PETRIS, S. (1981). Determination of equilibrium binding parameters of monoclonal antibodies specific for cell surface antigens. *Immunol. Meth.*, **2**, 1.

WAIBEL, R., O'HARA, C.J. & STAHEL, R.A. (1987). Characterization of an epithelial and a tumor-associated human small cell lung carcinoma antigen. *Cancer Res.*, **47**, 3766.

WAIBEL, R., O'HARA, C.J., SMITH, A. & STAHEL, R.A. (1988). A tumour-associated membrane sialoglycoprotein on human small cell carcinoma identified by the IgG2a monoclonal antibody SWA20. *Cancer Res.*, **48**, 4318.

ZIMMER, A.M., ROSEN, S.T., SPIES, S.M. & 4 others (1985). Radioimmunoimaging of human small cell lung carcinoma with I-131 tumor specific monoclonal antibody. *Hybridoma*, **4**, 1.